# Development of a large-scale rapid LAMP diagnostic testing platform for pandemic preparedness and outbreak response

**DOI:** 10.1093/biomethods/bpae090

**Published:** 2024-11-27

**Authors:** Rinie van Beuningen, Kin Ki Jim, Maikel Boot, Michel Ossendrijver, Bart J F Keijser, Jeroen H B van de Bovenkamp, Willem J G Melchers, Tim Kievits

**Affiliations:** Stichting Therapie op Maat (TOMi Foundation), 's-Hertogenbosch, 5223DE, The Netherlands; Department of Fundamental Microbiology, Faculty of Biology and Medicine, University of Lausanne, Lausanne, CH-1015, Switzerland; Microbiology and Systems Biology, Netherlands Organisation for Applied Scientific Research (TNO), Leiden, 2333BE, The Netherlands; Microbiology and Systems Biology, Netherlands Organisation for Applied Scientific Research (TNO), Leiden, 2333BE, The Netherlands; Microbiology and Systems Biology, Netherlands Organisation for Applied Scientific Research (TNO), Leiden, 2333BE, The Netherlands; Eurofins PAMM Laboratorium Medisch Microbiology, Veldhoven, 5504DL, The Netherlands; Department of Medical Microbiology, Radboud University Medical Center, Nijmegen, 6525AJ, The Netherlands; Stichting Therapie op Maat (TOMi Foundation), 's-Hertogenbosch, 5223DE, The Netherlands

**Keywords:** LAMP, COVID-19, pandemic preparedness, SMART-LAMP, SARS-CoV-2

## Abstract

The coronavirus disease 2019 (COVID-19) pandemic underscored the necessity for rapid and efficient diagnostic testing to mitigate outbreaks and control disease transmission. While real-time reverse transcriptase quantitative PCR (RT-qPCR) has been the gold standard due to its high sensitivity and specificity, its logistical complexities and extended turnaround times highlighted the need for alternative molecular methods and non-standard equipment and consumables not subject to supply chain pressure. Loop-mediated isothermal amplification (LAMP) offers several advantages over RT-qPCR, including faster processing time, assay flexibility and cost-effectiveness. During the pandemic, LAMP was successfully demonstrated as a viable alternative to RT-qPCR for SARS-Related Coronavirus 2 detection. However, due to a 100 to 1,000-fold increase in testing volumes, there was an imminent need for automating and scaling up existing LAMP testing workflows leveraging a robotic infrastructure, while retaining analytical performance and cost-effectiveness. In 2020, the Foundation TOMi started the “TOMi corona initiative” to develop and validate a high-throughput, end-to-end, automated, scalable single-step RNA purification, and LAMP-based COVID-19 testing system called SMART-LAMP (Scalable Molecular Automation for Rapid Testing using LAMP) that can process up to 40,000 samples per day using existing laboratory equipment infrastructure with sensitivity comparable to RT-qPCR. This system provides a rapid and scalable diagnostic solution for future pandemics, capable of processing over 40,000 samples per day. In addition, the system is designed to minimize consumable costs and reduces the overall use of plastics to align with increasingly strict sustainability goals that will be imposed over the coming years. Importantly, this system and public–private partnerships in the TOMi corona initiative has the potential to serve as a baseline to enhance pandemic preparedness and response capabilities.

## Introduction

The coronavirus disease 2019 (COVID-19) pandemic has highlighted the importance of efficient and rapid diagnostic testing in pandemic preparedness and outbreak response. Accurate and fast diagnostic tests are essential for controlling outbreaks and for identifying and isolating infected individuals, thereby reducing pathogen transmission [1–3]. Throughout the pandemic, the predominant method for diagnostic testing of the SARS-Related Coronavirus 2 (SARS-CoV-2) virus has been real-time reverse transcriptase quantitative PCR (RT-qPCR). RT-qPCR has high sensitivity and specificity and typically provides results within 24 hours, but this time window is often extended due to logistical complexities, such as sample transport between testing sites and laboratories. In addition, traditional RT-qPCR methods are seen as complex and relatively expensive. Despite the effectiveness and accuracy of RT-qPCR, the high demand for testing and shortages of consumables (e.g. reagents and plasticware) during the early stages of the pandemic had a severe impact on laboratory capacities and municipal health services, resulting in delays and underscoring the urgency to enhance testing capabilities and accelerate the diagnostic surveillance.

During the COVID-19 pandemic, alternative methods (e.g. rapid antigen-based testing and Loop-mediAted isotherMal amPlification (LAMP)), have been developed to increase the speed from sampling to result, to scale up testing, and to reduce the reliance on RT-qPCR [4, 5]. Rapid antigen-based testing offers a fast turnaround time limited with regard to efficiency in execution and the sensitivity is generally lower compared to RT-qPCR [4, 6]. However, the usual development time costs several months after the initial discovery of a pathogen, rendering it less useful for rapid response to an outbreak. As an alternative to RT-qPCR, nucleic acid amplification techniques like LAMP, developed by Eiken Chemical Co. over two decades ago, can be employed [7, 8]. Similar to PCR, LAMP allows the specific amplification of target DNA using primers specific to the target region. Different from PCR, LAMP operates at a constant temperature, typically between 60 and 70°C. When detecting RNA viruses, such as SARS-CoV-2, the viral RNA must first be converted to cDNA via a reverse transcriptase step. In this process, the reverse transcriptase and DNA polymerase work simultaneously within a single-tube reaction. The technique uses 4–6 primers, with two (FIB/BIP) being hybrids that target two different gene regions. A key feature of the LAMP reaction is the formation of a dumbbell structure, which allows for rapid exponential amplification and the creation of repeating DNA sequences. This ensures both fast and highly sensitive detection of target sequences. LAMP has demonstrated robustness against impurities in reaction mixtures, allowing for more efficient sample pre-processing [9]. Perhaps more importantly, because LAMP typically does not require thermocyclers, it is considered more cost-effective and can be adapted and deployed to resource-limited, point-of-care, or field-use settings. These characteristics make LAMP particularly attractive for various diagnostic applications and across high- and middle-income countries.

Since its inception, LAMP has been widely used across different pathogens (e.g. bacterial, viral and fungal) and biological matrices (e.g. swabs and saliva), showcasing its applicability to public health threats and epidemic-scale outbreaks [10–12]. As such, also during the COVID-19 pandemic, several groups successfully demonstrated that LAMP was a viable alternative to RT-qPCR [13–17]. While building a reliable and robust LAMP diagnostic workflow is well manageable at low volumes of testing, with three orders of magnitude differences in scale and volume requirements (i.e. 100,000 tests instead of 100 per day) during the COVID-19 pandemic, scaling and automating LAMP-based workflows, while retaining their analytical performance became a major challenge. Over the course of the pandemic, there have been several initiatives that have shown proof-of-concept scaling to 1000–10,000 molecular tests a day, achieved through automation and robotics [18–23]. However, these workflows are typically revolving around RT-qPCR workflows that oftentimes require a large upfront capital investment, and high consumable and reagent costs. Additionally, these workflows have a turnaround time that is about 5-fold higher than a LAMP-based assay. Notably, while some programs have demonstrated the ability to process over 40 000 samples daily, the majority of these workflows remain limited to scaling up to 10,000 samples per day [24–27]. In the light of national screening in the Netherlands, would be insufficient to meet pandemic testing demands.

To meet the >10,000 per day requirements for daily testing in the Netherlands, the TOMi Foundation (‘s-Hertogenbosch, The Netherlands) received funding from the Dutch government to start the TOMi corona initiative and together with Stichting PAMM (Veldhoven, the Netherlands), Radboud University Medical Center (Nijmegen, The Netherlands), Pivot Park Screening Center (Oss, The Netherlands), the Dutch municipal health services (GGD) and Netherlands Organisation for Applied Scientific Research (TNO) to develop and validate a large-scale rapid molecular diagnostic system based on LAMP for future pandemics or outbreaks. The TOMi corona initiative stretches beyond the automated workflow outlined in this manuscript and also includes infrastructure for logistics, consumables, data transfer and a comprehensive group of partners and key opinion leaders.

The infrastructure described in this article was built based on an existing automated infrastructure equipped with advanced robotic systems for high-throughput drug screening (Pivot Park Screening Centre). By integrating single-step, 96-well centrifuge-based RNA purification with LAMP detection leveraging 96-well sample tubes and barcoded 96-wells plates, testing up to 40 000 samples per day with a limit of detection similar to that of RT-qPCR but significantly faster than routine RT-qPCR platforms can be achieved. In conclusion, this system, called SMART-LAMP, can potentially enhance pandemic preparedness and response capabilities by providing a robust, rapid, and scalable diagnostic tool.

## Materials and methods

### RNA purification

The BioEcho EchoLUTION^TM^ viral RNA/DNA kit was used for RNA purification, enabling fast RNA purification in a single centrifuge step. For spiked samples and evaluation panel samples, 45 µL of the test sample was added to 45 µL LyseNtact lysis buffer (EchoLUTION^TM^, BioEcho) and transferred to 1 mL dual 2D/1D barcoded LI1000 tubes (LVL Technologies). These 2D/1D tubes have a 2D barcode for automation while also having a 1D linear barcode and readable ID, which is important for the sample processing step. Patient samples (e.g. nasopharyngeal swabs) can be mixed directly with 500 µL LyseNtact lysis buffer in 1 mL barcoded tubes. After scanning each tube’s barcode, the tubes were placed in a barcoded 96-tube rack. Upon transfer of the racks to the RNA purification site at the PPSC (Oss, The Netherlands) the 2D barcoded racks and 96 1 mL tubes were scanned. These racks (max. 4 per centrifugation run) were spun down at 1,500 RPM for 3.5 min followed by decapping using an automated tube decapper (SAFE^®^ 96-channel capper/decapper IT, LVL technologies). The racks were then placed on a Biomek liquid handling platform (Biomek FX liquid handler, Beckman Coulter) and 90 µL (spiked samples and evaluation panel samples) or 100 µL (patient samples) of the lysate was transferred to a 96-well BioEcho RNA purification plate respectively for single-step, easy to use and high speed, RNA purification using a 96-tip-head of the liquid handling platform. These plates were spun down at 1500 RPM for 3.5 min to obtain 20-25 µL ready-to-use RNA solution and placed back on the liquid handling platform. The open tubes in the 96-tube racks were capped using clean caps with a semi-automated 96-channel capper (SAFE^®^ Cap Seal semi-automatic, LVL technologies). Then, 10 µL of the RNA solution was transferred using the 96-tip-head of the liquid handling platform to a black polypropylene flat bottom 384-well plate (Greiner cat. No. 781209) for the LAMP amplification reaction. All equipment and reagents used in this study are described in Supplementary Tables S1 and S2.

### LAMP-based detection

The primer sequences used were adapted from a previously published study [5, 28] and are shown in Supplementary Table S3. For the fluorescent LAMP reaction, a volume of 25 μL was used. A contactless liquid dispenser (CERTUS FLEX, Gyger) was firstly used to add 15 μl of a premixed solution containing 12.5 μL WarmStart LAMP 2x Master Mix (NEB), 1.5 μL primer stock containing all six LAMP primers (B3, F3, LB, LF, FIP, BIP) for the viral target (Orf1ab gene), 0.5 μL Syto9 (1 μM final, 50 μM stock), and 0.5 μL guanidine-HCl (40 mM final, 2 M stock) and second, 10 μL of mineral oil M8662-5VL (Merck Life Sciences) to each well of the 384-well plates containing the 10 μL RNA solution followed by plate sealing with a thermal microplate sealer (PlateLoc, Agilent). Guanidine was added to enhance the speed and sensitivity of the LAMP reaction [29]. The mineral oil provides an additional barrier to prevent contamination in case the sealing is not perfect during the amplification and disposal of amplified plates. Furthermore, 0.5 μL guanidine-HCl (40 mM final, 2 M stock) was only added to the Orf1ab reaction and not to the RNaseP control reaction. Primers were used at final concentrations of 0.2 μM for F3/B3, 0.4 μM for LB/LF and 1.6 μM for FIP/BIP. Each plate was incubated at 65°C for 25 minutes using a Cold Plate Air Cooled Heater/Cooler (CPAC Ultraflat HT 2-TEC, Inheco) equipped with a custom-made adapter for 384-well flat bottom plates (Inheco) for optimal heating kinetics of the whole plate and a top heater. Customization of the CEPAC heater involved tailoring the adaptor to more tightly fit the assay plates and flattening the crew with which the plate was attached to the device to ensure optimal heat transfer. The top heater provides faster heating but limits access by a robot gripper in the Biomek liquid handling platform or the large robots used at PPSC. We therefore also worked with the addition of 10 μL mineral oil to prevent condensation in case we did not use the heated lids. After incubation, fluorescent data was collected with a microplate reader (Pherastar FSX, BMG Labtech) using an excitation wavelength of 485 nm and an emission wavelength of 520 nm. The calculation method automatically distinguishes between positive and negative signals using a 3-step process:

- Order the fluorescent signals (S): Arrange all the samples' fluorescent signals from highest to lowest, forming a sequence *S*_1_, *S*_2_, *S*_3_, …, *S*_last_. This also included data from the positive (PC) and negative controls (NC).
- Calculate the relative delta (Δ*S_n_*): For each sample in the ordered list (except the last one), calculate the relative delta as follows: Δ*S_i_* = (*S_i_* - *S_i_*_+1_)/*S_i_*
- Determine the cutoff point: The cutoff between positive and negative samples is identified by finding the maximum relative delta Δ*S_i_*. All samples from *S*_1_ to *S_i_* are considered positive and samples from *S_i_*_+1_ to *S*_last_ are considered negative.

This method can be applied to any plate reader and eliminates the need for a predefined cut-off, requiring only a set of PC and NC.

### Thermal profiling in 384-well flat bottom plates during LAMP

Depending on the disposables used, heating kinetics issues can arise in LAMP and PCR assays, including inconsistent temperature uniformity, edge effects, variable heating rates, and thermal lag [7–9]. These factors can lead to discrepancies in reaction kinetics and efficiency across wells. Due to the need for fast and accurate temperature cycling, PCR-based assays use v-shaped plates or tubes. Handling of these plates by robot arms and grippers, sealing and fluorescent read-out through the top of these plates is however challenging. We therefore optimized the incubator to work with sturdy, easy-to-seal and fluorescently read flat-bottom plates. To assess the heating kinetics in the 384-well flat bottom plates, we measured the temperature of single wells in a 384-well plate at different positions across the plate (wells A1, A13, L1, L13) with a thermocouple (RS PRO RS42, RS Components, Haarlem, the Netherlands) over a period of 15.5 minutes, with an interval of 30 seconds (Supplementary Fig. S1).

### Positive and negative controls

As a PC, 20 µL of NATtrol™ SARS-CoV-2 External Run Control (6 × 0.5 mL) (CATALOG# NATSARS(COV2)-ERC, Zeptometrix), containing 50 000 SARS-CoV-2 RNA copies per mL, was added in duplicate to each bioEcho RNA purification run. This results in each PC containing 1000 SARS-CoV-2 RNA copies per sample. As a NC, 20 µL of NEG: NATtrol™ SARS-Related Coronavirus 2 (SARS-CoV-2) External Run Control (6 × 0.5 mL) (CATALOG# NATSARS(COV2)-NEG, Zeptometrix), containing 50 000 human A-549 cells per mL, was added in duplicate to each bioEcho RNA purification plate. This results in each NC containing 1000 lysed human A-549 cells per RNA purification. Subsequently, 1/4 of the respective RNA solution is used as input for the LAMP assay, corresponding to 250 SARS-CoV-2 RNA copies or RNA isolated from 250 human A-549 cells per LAMP reaction.

### High positive controls used for checkerboard assay

A high positive (HP) sample is prepared by pooling clinical samples that scored strongly positive (Cq ≤ 20) in SARS-CoV-2 PCR assays or using an appropriate standard containing a high quantity of SARS-CoV-2 RNA (e.g. the RIVM Positive Run Control, Cq ≤ 20). These HP samples were anonymized routine SARS-CoV-2 screening retention samples provided by the Stichting PAMM (Veldhoven, The Netherlands) laboratory and used for the sample-to-sample carryover/checkerboard analysis. RNA was isolated from a total volume of 125 µL from each HP with a MagNApure 96 (Roche) system resulting in an RNA solution of 50 µL and stored at −20°C until use.

### Sample-to-sample carryover (checkerboard) analysis

Two BioEcho RNA purification runs are conducted using a checkerboard layout (Supplementary Fig. S2) of alternating NC and HP samples to detect potential cross-contamination. In the second BioEcho RNA purification run, the positions of the NC and HP samples are swapped relative to the first run. The positions of the run controls are also changed. In total, 92 HP and 92 NC samples are placed (in addition to the run controls). The tests are then repeated in duplicate. Thus, a total of 184 HP samples and 184 NC samples were analyzed.

### Qnostics, QCMD, and RIVM positive run control SARS-CoV-2 test panel for sensitivity testing of the SMART-LAMP system

To verify and validate the SMART-LAMP system for SARS-CoV-2 testing, we used two commercially available verification panels, the Qnostics SARS-CoV-2 Analytical Q Panel (#SCV2AQPo1-A) and the External Quality Assessment (EQA) panel managed by the Quality Control for Molecular Diagnostics (QCMD, Glasgow, UK), and a SARS-CoV-2 positive run control panel from the RIVM. In total 45 μL of each sample from the validation panels was added to 45 μL of the lysis buffer. This mixture undergoes RNA purification using the BioEcho RNA purification method as previously described. From the purified RNA, 10 μL RNA solution is used for the LAMP amplification as described earlier.

### Retrospective validation of the SMART-LAMP system with clinical samples

For this part of the study, we used samples that have been tested previously with information on the Cq values. The positive samples are selected based on the obtained Rt-qPCR Cq result. Samples were selected per category based on the Cq values. Within categories, selection is not based on the Cq value but on the order of availability/date of collection. The samples were stored in the lysis buffer previously used for the reference RT-qPCR test. After thawing, 90 μL of this solution is used as input for the bioEcho RNA purification.

### Prospective validation of the SMART-LAMP system with clinical samples

The new test system introduces an alternative sample collection method using 1 mL 2D/1D barcode tubes containing 0.5 ml of BioEcho lysis buffer instead of using the standard 10 mL tube with 3 mL solution as employed in traditional SARS-CoV-2 testing protocols in the Netherlands. This innovative approach increased sensitivity 6-fold, while significantly enhancing both automation and logistics. With the BioEcho lysis buffer, we demonstrated that RNA remained stable in this lysis buffer for at least a week at room temperature, and importantly, the solution rendered the samples non-infectious. However, a limitation is that retrospective testing of the collected samples is not possible. Therefore, we collected clinical samples prospectively and specifically for this study. At Dutch municipal health services (GGD) testing locations or another site where people are tested for SARS-CoV-2, a portion of the collected material was used for this study. Directly after the standard processing of the collected swab (placing the swab in the buffer of the PCR reference test), the swab is removed from this test tube and placed in a 1 mL tube containing the LyseNtact lysis buffer. This way, the material that remains on the swab after standard processing (and/or present in the fluid absorbed by the swab) is used to test the new system. It should be noted that material obtained this way contains less SARS-CoV-2 sample material compared to the reference method. This method has previously been shown to be sufficiently suitable for validating an alternative method, provided this limitation is taken into account (this method was selected and discussed in collaboration with PAMM Veldhoven, and using this sample collection method does not impose any additional burden on the participant). For all materials used in this study, a routine reference RT-qPCR test is performed as used at the testing location. Samples with discrepancies in results are retested with the reference PCR method targeting the coronavirus RdRP- and E-gen [30, 31] at the Radboud University Medical Center to confirm the initial result. For this retest, material from test tube 2 (tube filled with LyseNtact lysis buffer) is used to exclude that the difference in results is due to insufficient sample transfer.

### Ethical statement

The samples used in this study were originally collected by the Municipal Public Health Service Brabant-Zuidoost (GGD BZO) as part of routine COVID-19 diagnostic procedures. As this research involved anonymized residual samples from routine COVID diagnostics that were not specifically collected for research purposes, informed consent was waived in accordance with the Dutch Medical Research Involving Human Subjects Act (WMO). The study was conducted in line with the ethical principles of the Declaration of Helsinki, ensuring respect for the rights and confidentiality of the individuals from whom the samples were originally obtained.

## Results

### SMART-LAMP system

#### Workflow SMART-LAMP system

**Figure 1.**
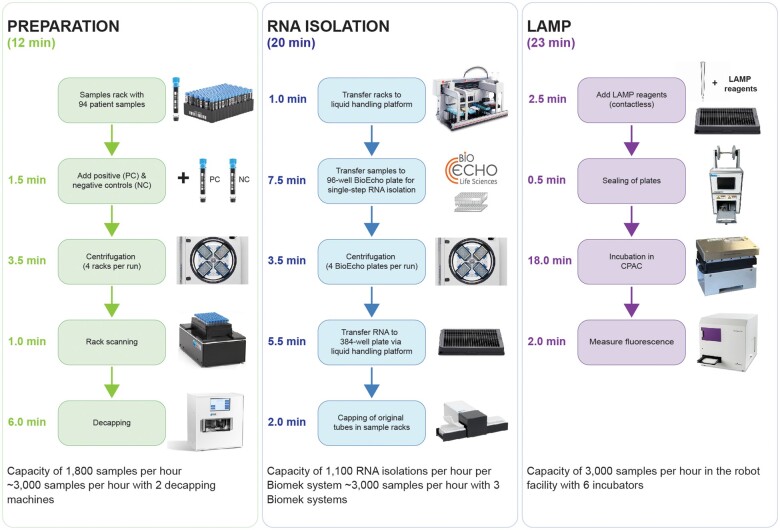
Schematic overview of the steps in the SMART-LAMP workflow.

**Figure 2.**
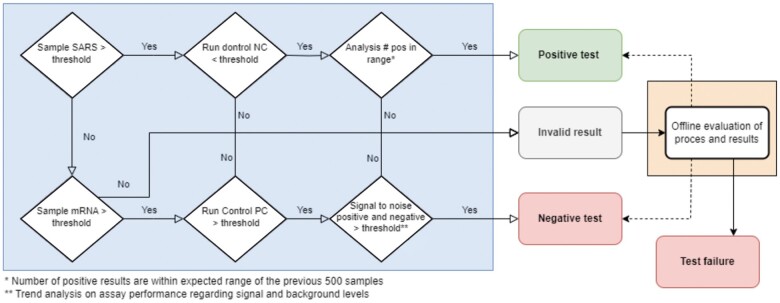
Workflow of data processing.

A workflow for high throughput LAMP-based detection of SARS-CoV-2 was established based on an existing robotic infrastructure for ultra High Throughput drug Screening (uHTS) at the Pivot Park Screening Centre (PPSC, Oss, The Netherlands). Equipment was converted and deployed to enable the workflow for large-scale rapid LAMP diagnostic testing. The workflow comprises three major steps: (1) sample preparation, (2) single-step RNA purification (3) LAMP amplification and detection (Fig. 1), and (4) data processing (Fig. 2). The set-up times, process times and samples per hour at PPSC can be found in Fig. 1. Leveraging 3 Biomek pipetting robots over the course of a 16-h shift led to a total of 16 × 2,500 samples = 40,000 samples per day with this workflow.

##### Step 1. Preparation of samples

Samples collected from patients (e.g. nasopharyngeal swabs) are put directly into a 2D barcoded 1 mL tube (LVL Technologies) prefilled with 500 µL lysis buffer (LyseNtact, BioEcho) and placed in a 96-tube custom rack. Per 92 samples, two positive (PC) and two negative batch control (NC) samples are added, resulting in a total number of 96 individual 2D barcoded tube samples per rack. Each rack has a unique barcode for later identification. Four racks (*n* = 368 samples) are then placed in a centrifuge and briefly spun down to prevent bioaerosols and spillage. After centrifugation, each rack and tube is scanned and transferred to an automated tube decapper SAFE^®^ capper 96 channel XT (LVL technologies) to remove the caps. The total estimated time of this step is 12 minutes with a capacity of about 1,800 samples per hour, which can be increased to about 2,800 samples per hour with 2 tube decappers.

##### Step 2. RNA purification

The racks with opened tubes are then placed on a liquid handling platform (Biomek FX, Beckman Coulter) and 100 µL of each sample or 100 µL control sample per rack is transferred to a 96-well BioEcho purification plate according to the layout of the original sample plate. The BioEcho platform allows fast and efficient high-quality RNA purification in a single-step purification and is suitable for high-throughput molecular diagnostic testing as compared to traditional magnetic beads RNA purification method such as described in Levison *et al.* 1998 [19]. RNA from the samples of the four plates is purified by a single centrifugation step for 3.5 minutes. From every well of the RNA solution plate, 10 µL of the purified RNA is transferred to each of the two identical 384-well flat bottom plates for LAMP assay; one for performing the SARS-CoV-2 LAMP assay, the other for performing the human RNaseP control LAMP assay. The remaining decapped tubes from the first step are recapped with a tube capper (SAFE^®^ Cap Seal semi-automatic, LVL technologies) and stored at 4°C. With one Biomek liquid handling platform, about 930 RNA purifications can be processed per hour, which can be increased to about 2,800 samples per hour with 3 Biomek liquid handler platforms. The total estimated time of this step is 20 minutes.

##### Step 3. LAMP amplification

The LAMP reagents, containing the LAMP master mix, primers for either the viral or the human control target, with a total volume of 25 μL are added to each well of the 384-well plates containing 10 μL RNA solution with a contactless liquid dispenser (CERTUS FLEX, Gyger). The plates are then sealed with a thermal microplate sealer (PlateLoc, Agilent), and placed onto a CPAC plate heating device (CPAC Ultraflat HT 2-TEC, Inheco) equipped with a custom-made adapter for 384-well flat bottom plates (Inheco), and incubated at 65°C for 23 minutes. To ensure appropriate heating conditions, the heating kinetics across different wells were determined and evaluated for their effects on the LAMP assay (Supplementary Table S4). After incubation, fluorescent data measurements are performed in a microplate reader (Pherastar FSX) with an excitation wavelength of 485 nm and an emission wavelength of 520 nm. The total estimated time of this step is 23 minutes with a capacity of about 2,500 samples per hour with six CPAC heating devices.

##### Step 4. Data processing and result classification

The classification of test results was based on an automated analysis of fluorescence data from both the viral target (SARS) and human control (mRNA). The data processing workflow aimed to distinguish between positive, negative, invalid, and failed test results through a series of quality control steps, as shown in the decision-making flowchart (Fig. 2).

- Threshold Checks: The analysis began by checking if the SARS signal exceeded a defined threshold. If not, the mRNA signal was checked. If the mRNA signal exceeded the thresholds, the sample was classified as “Negative.”
- Control Evaluation: For samples above the thresholds, the NC and PC were assessed to confirm assay performance. If these controls did not meet expected criteria, the result was marked as “Invalid.”
- Trend Analysis: The number of positives was compared to an expected range based on recent trends (last 500 samples). If within range, the result was classified as “Positive.”
- Signal-to-Noise Check: If the positive count was outside the expected range, the signal-to-noise ratio was evaluated. A ratio above a set threshold led to a “Negative” classification; otherwise, “Offline Evaluation” was required.
- Offline Evaluation: Results that could not be automatically classified underwent further review. If issues were identified, the test was marked as a “Test failure.”

This workflow ensured accurate classification by dynamically adjusting thresholds and including quality control at multiple stages.

### Robustness of detection of SARS-CoV-2 RNA and checkerboard analysis

To evaluate the robustness of SARS-CoV-2 RNA detection, we first tested 50 PC samples, each spiked with 1,000 copies of SARS-CoV-2 RNA in the SMART-LAMP system. All 50 samples (100%) tested positive, confirming the system's effectiveness. To rule out cross-contamination we performed a checkerboard analysis with alternating NC and HP samples in two BioEcho RNA purification runs. In the second BioEcho purification run, the positions of the NC and HP samples are swapped relative to the first run, without changing the positions of the run controls. The experiment was performed in duplicate and in total 184 HP samples and 184 NC samples were analyzed and showed that all NC samples were negative for SARS-CoV-2 RNA, and all HP samples were positive for SARS-CoV-2 RNA.

### SARS-CoV-2 analytical sensitivity testing

To evaluate the analytical sensitivity of the SMART-LAMP system for detecting SARS-CoV-2, we conducted six independent runs using a 2-fold serial dilution of a PC provided by RIVM. The dilution series ranged from 2,000 to 8 gene copies per reaction, with a blank sample included as a control. Each of the eight dilutions was tested in quintuplicate (five replicates per dilution) across all runs. We compared LAMP amplification performance between the CPAC heating device and a commercially available 96-well RT-qPCR machine (CFX Connect RT-qPCR cycler, Bio-Rad). The results are presented in Table 1. Both setups consistently achieved a 100% detection rate at higher concentrations, from 200,000 copies per mL down to 6250 copies per mL, indicating robust performance of the SMART-LAMP system at elevated viral loads. As the concentration decreased, the detection rate began to decline. At 3125 copies per mL, the CFX device showed a reduced detection rate of 90%, while the Inheco system maintained 100% detection. This suggests that the Inheco heating system may offer slightly better sensitivity at this concentration. The detection rates continued to decrease at lower viral concentrations. At 1562 copies per mL, the percentage of positive detections dropped to 83.3% for the Inheco and 70% for the CFX. At the lowest concentration tested (781 copies per mL), detection rates fell further to 63.3% for the Inheco and 53.3% for the CFX, indicating a notable decline in sensitivity at these lower levels. To validate these findings, we used a commercially available SARS-CoV-2 analytical panel (Qnostics, SARS-CoV-2 Analytical Q Panel, #SCV2AQPo1-A), which includes nine samples covering the dynamic range of SARS-CoV-2 infection. Four replicates per sample were tested, and the results are detailed in Supplementary Table S5. Both the CPAC heating device and the CFX cycler consistently detected concentrations of at least 5,000 copies per mL. The CPAC incubator demonstrated slightly better sensitivity, with a detection threshold as low as 500 RNA copies per mL (equivalent to 7.5 RNA copies per LAMP assay), compared to the CFX.

**Table 1.** Evaluation of the analytical sensitivity.

Sample	Copies/mL	Copies/µL	Copies/reaction	Incubator	
				CPAC	CFX
1	200,000	200	2000	30/30	30/30
2	100,000	100	1000	30/30	30/30
3	50,000	50	500	30/30	30/30
4	25,000	25	250	30/30	30/30
5	12,500	12.5	125	30/30	30/30
6	6,250	6.3	63	30/30	30/30
7	3,125	3.1	31	30/30	27/30
8	1,562	1.6	15	25/30	21/30
9	781	0.8	8	19/30	16/30
Blank	0	0	0	0/4	0/4

Detection rates (%) for the SMART-LAMP system using CPAC and CFX devices, tested across serial dilutions of a SARS-CoV-2 run control (RIVM) from 200,000 to 781 copies/mL in quintuplicate over six independent runs. Both devices showed 100% detection at higher concentrations (200,000–6,250 copies/mL), with decreasing sensitivity at lower concentrations, particularly below 3,125 copies/mL.

### External quality assessment panel and RIVM PC panel

To further validate the SMART-LAMP system, we tested SARS-CoV-2 samples from the External Quality Assessment (EQA) panel of Qnostics managed by the Quality Control for Molecular Diagnostics (QCMD) and the RIVM PC panel with the results shown in Table 2A and B, respectively. The CPAC heating device showed a good performance with the EQA panel of Qnostics with a sensitivity of about 900 RNA copies per mL (14 RNA copies per LAMP assay) and the RIVM PC panel (national standard) with a sensitivity of about 1,500 RNA copies per mL (16 RNA copies per LAMP assay).

**Table 2.** Performance of the SMART-LAMP System with external evaluation quality panels.

A			
Sample	Copies/mL	Incubator	
		CPAC	CFX
**1**	1,000,000	4/4	4/4
**2**	100,000	4/4	1/4
**3**	Negative	0/4	4/4
**4**	5,000	4/4	2/4
**5**	1,000	2/4	0/4

B					
Sample	Copies/mL	Copies/µL	Copies/reaction	Incubator	
				CPAC	CFX
1	200,000	200	2,000	4/4	4/4
2	100,000	100	1,000	4/4	4/4
3	50,000	50	500	4/4	4/4
4	25,000	25	250	4/4	4/4
5	12,500	12.5	125	4/4	2/4
6	6,250	6.3	63	2/4	3/4
7	3,125	3.1	31	0/4	0/4
8	1,562	1.6	15	0/4	0/4
9	781	0.8	8	0/4	0/4
Blank	0	0	0	0/4	0/4

The table presents sensitivity testing of the SMART-LAMP system for SARS-CoV-2 detection, comparing the Inheco incubator and CFX platform across two sections: A: Results using the External Quality Assessment (EQA) panel from Qnostics, managed by the Quality Control for Molecular Diagnostics (QCMD), section, B: Results using the RIVM control panel.

### Retrospective validation of the SMART-LAMP system with clinical samples

To assess the performance of the SMART-LAMP system with clinical samples, a retrospective validation was carried out using 200 known positive and 200 negative samples from routine SARS-CoV-2 screening (Stichting PAMM, Veldhoven, The Netherlands). Positive samples were categorized based on their RT-qPCR cycle threshold (Ct) values into five categories (Table 3). The results showed a high detection rate in the lower Cq categories: all 17 samples in CAT 1 (Ct < 20) were correctly identified as positive (100% detection rate), and 78 out of 80 samples in CAT 2 (Ct 20–30) were detected as positive, yielding a detection rate of 98%. These results met the predefined acceptance criteria of 100% and >95% detection, respectively. However, the detection rate declined in higher Cq categories, indicating lower viral loads. In CAT 3 (Ct 31–35), 22 out of 36 samples were detected as positive (61% detection rate), falling short of the >80% acceptance criterion. In CAT 4 (Ct 36–40), the detection rate dropped further, with only 15 out of 52 samples testing positive (29% detection), below the >50% criterion. For CAT 5 (Ct > 40), representing very low viral loads, only 2 out of 15 samples were detected as positive (13% detection). For the negative samples, all 189 were correctly identified as negative, achieving 100% specificity, which met the acceptance criterion of <5% false positives. Overall, the SMART-LAMP system demonstrated excellent performance for samples with higher viral loads (CAT 1 and CAT 2) and for negative samples, meeting the predefined criteria. However, sensitivity decreased significantly in samples with higher Cq values (CAT 3 and CAT 4), suggesting limitations in detecting low viral load cases.

**Table 3.** Retrospective validation of the SMART-LAMP system using clinical samples.

A			
Category	Description	Results	Score (%)
CAT 1	Cq <20	17/17 positive	100
CAT 2	Cq 20–30	78/80 positive	98
CAT 3	Cq 31–35	22/36 positive	61
CAT 4	Cq 36–40	15/52 positive	29
CAT 5	Cq >40	2/15 positive	13
Negative	Negative	0/189 positive	100

B					
		SARS-CoV-2 PCR			
		POS	NEG	Cq >40	Total
**SARS-CoV-2 LAMP**	**POS**	132	0	2	134
	**NEG**	53	189	13	255
	**Total**	**185**	**189**	**15**	**389**

A. LAMP performance per Cq range. B. Comparison LAMP versus RT-qPCR.

### Prospective validation of the SMART-LAMP system with clinical samples

For prospective validation of the SMART-LAMP system, Stichting PAMM laboratory selected 621 clinical samples (101 positive and 520 negative) of which the Cq scores were unblinded after the release of the SMART-LAMP test results to Stichting PAMM laboratory. The 101 positive samples are selected based on the previously determined RT-qPCR Cq result and assigned to one of 5 categories or the negative group based on the Cq value (Table 4A). As shown in Table 4B, there was a 93% concordance between the LAMP and RT-qPCR. The 10 false-negative samples were confirmed false negatives by retesting by RT-qPCR.

**Table 4.** Prospective validation of the SMART-LAMP system using clinical samples.

A			
Category	Description	Results	Score (%)
CAT 1	Cq <20	5/5 positive	100
CAT 2	Cq 20–30	73/76 positive	96
CAT 3	Cq 31–35	9/13 positive	69
CAT 4	Cq 36–40	4/7 positive	57
CAT 5	Cq >40	0/3 positive	0
Negative	Negative	8/515 positive	1.55

B					
		SARS-CoV-2 PCR			
		POS	NEG	Cq >40	Total
**SARS-CoV-2 LAMP**	**POS**	93	8	0	101
	**NEG**	10	507	3	520
	**Total**	**103**	**515**	**3**	**621**

A. LAMP performance per Cq range. B. Comparison LAMP versus RT-qPCR.

## Discussion

In this article, we provide a methodological blueprint, proof-of-concept and clinical validation that shows that a single-step RNA purification in combination with the LAMP assay for SARS-CoV-2 can be successfully adopted in a scalable, highly automated high-volume molecular lab unit that is ideally suited for the specific demands under pandemic crisis. While we acknowledge that certain optimization steps are required to further deploy and scale the system, this blueprint can pave the way for global pandemic preparedness programs and offer a scalable molecular diagnostics workflow that can be used during future epidemic or pandemic settings.

### LAMP as a viable qPCR alternative

The LAMP amplification procedure is an attractive method for molecular diagnostics, complementing PCR-based tests. As it is an isothermal assay, the LAMP method does not require a thermocycler and can be performed in various thermal incubators. The isothermal nature of the assay eliminates the need for PCR plates to perform the assay, allowing for the use of a more diverse array of reaction plates. This can be important as we have seen during the pandemic where plasticware for PCR tests became a scarce commodity. At the same time, this method ensures fast and efficient amplification of target sequences, resulting in a rapid and sensitive detection of viral and other pathogens. Since the method deploys up to eight specific primers per gene target, LAMP has a high specificity. Furthermore, this method is relatively robust towards impurities in the reaction mixture, allowing simplified sample handling and purification procedures.

Like all molecular tests, LAMP has its challenges, limitations, and areas for improvement. Analytical panel testing showed that the workflow performed well and met the required standards for SARS-CoV-2 detection. However, validation with clinical samples revealed that, while there was high concordance with RT-qPCR for samples with low Cq values (high viral loads), the sensitivity declined for samples with higher Cq values (30–40), indicating potential limitations in detecting low viral loads, or possible residual inhibition in using clinical samples. A possible improvement can be accomplished when avoiding the UTM transport medium, switching to a molecular transport medium that lyses and stabilizes viral RNA and is optimally aligned with molecular workflows. Furthermore, compared to RT-qPCR, LAMP has been shown to be prone to a higher false positivity rate, especially after prolonged amplification times, with improper assay designs or suboptimal buffer and reaction conditions. To reduce this, different strategies for improvement have been proposed, introducing sequence-specific probes (DARQ) as well the application of CRISPR-based detection of LAMP amplified products [32–34] other strategies have implemented sequence-based detection [27, 35]. However, the latter would require collecting materials after amplification, imposing significant risk for contamination. It is studies like these that are at the core of improving high-volume LAMP-based workflows for high-volume testing when all of these issues amplify with orders of magnitude because of the number of tests performed.

### Expandability of the SMART-LAMP system to other pathogens and biological materials

LAMP has demonstrated significant versatility and adaptability in its application to a wide array of pathogens and biological matrices [12–17]. This versatility is evidenced by the application of LAMP to saliva samples, which streamlines the collection process and obviates the need for specialized consumables, thereby rendering the procedure both cost-effective and automation friendly [11, 36]. Combined with innovations in robotic automation to accommodate other biological matrices, this makes the SMART-LAMP system a versatile LAMP-based platform that can be modified accordingly. In addition, the proposed system infrastructure could also be leveraged for any other isothermal molecular assays in the future.

In this current study, a direct head-to-head clinical validation was not conducted as it was deemed to be too intrusive on the participants to provide two nasal swabs and obtaining quick ethical approval for such a study was not possible at the time of the studies. However, further development and clinical validation of the SMART-LAMP system in accordance with the applicable ISO13485, ISO15189 and IVDR guidelines would be needed to prepare a fit-for-purpose SMART-LAMP platform that serves as a starting point during the next outbreak, ready-to-be-tuned, and made specific for each new pandemic pathogen.

### High-volume-based and cost-effective testing through the SMART-LAMP system

LAMP as a technique and assay itself is devoid of complex and costly equipment, and leverages readily available reagents. During the COVID-19 pandemic, LAMP-based tests emerged as an affordable and accessible alternative to qPCR, facilitating rapid and large-scale testing initiatives. During this time, there have been several initiatives that have shown proof-of-concept scaling up between 1000 and 5000 molecular tests a day, achieved through automation and robotics [18–22]. Notably, Lou *et al*. set up a gargling-based protocol that averaged a 6-h turnaround time with great cost-effectiveness. In that study, the economic efficiency of LAMP has been estimated to cost around 5 euros per sample when conducting around 10 000 tests a day [22]. Since the cost can be calculated in many different ways (cost of goods, cost to customer, raw material cost) comparisons are difficult to make. However, initial calculations conducted as part of the TOMi corona initiative show a comparable cost structure that scales with the volume tested. Cost can be further reduced with negotiated consumable and reagent contracts. Currently, SMART-LAMP leverages single-step BioEcho RNA purification plates that are designed to reduce costs by 59% [37] compared to silica-based methods. Besides the costs, the SMART-LAMP workflow also provided a significant reduction in the use of laboratory plastics. The protocol as it is written, uses a minimal amount of only 2 pipet tips per sample. In a time where sustainability is moving to the center stage, and laboratory consumables can become scarce under conditions of a pandemic crisis, we think this is a highly important aspect for a high-volume methodology such as the one presented in this study. Cost reduction can be further optimized in the future, with integration of a multiplex assay or pooled sampling approach.

## Supplementary Material

bpae090_Supplementary_Data
