# Supplementary material for: Development of a large-scale rapid LAMP diagnostic testing platform for pandemic preparedness and outbreak response
Source: Biol Methods Protoc. 2024 Nov 27;9(1):bpae090. doi: 10.1093/biomethods/bpae090 (PMC11634539; doi:10.1093/biomethods/bpae090)
Supplement: bpae090_Supplementary_Data [file bpae090_supplementary_data.docx]

**Supplemental Figures**

**Supplemental Figure 1. Temperature profiles flat bottom plate wells.** No significant differences were observed between the wells and a temperature of 63°C was reached after 6:30 minutes, 64°C after 7:30 minutes and 65°C after 9 minutes.

**
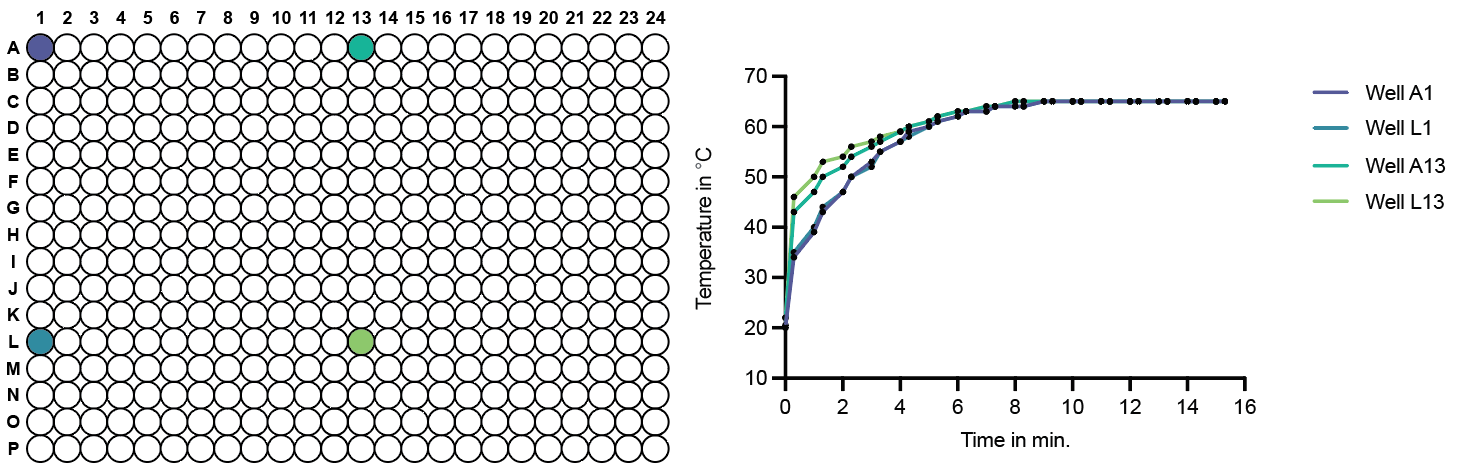
**

**Supplemental figure 2. BioEcho RNA purification run checkerboard layout.**


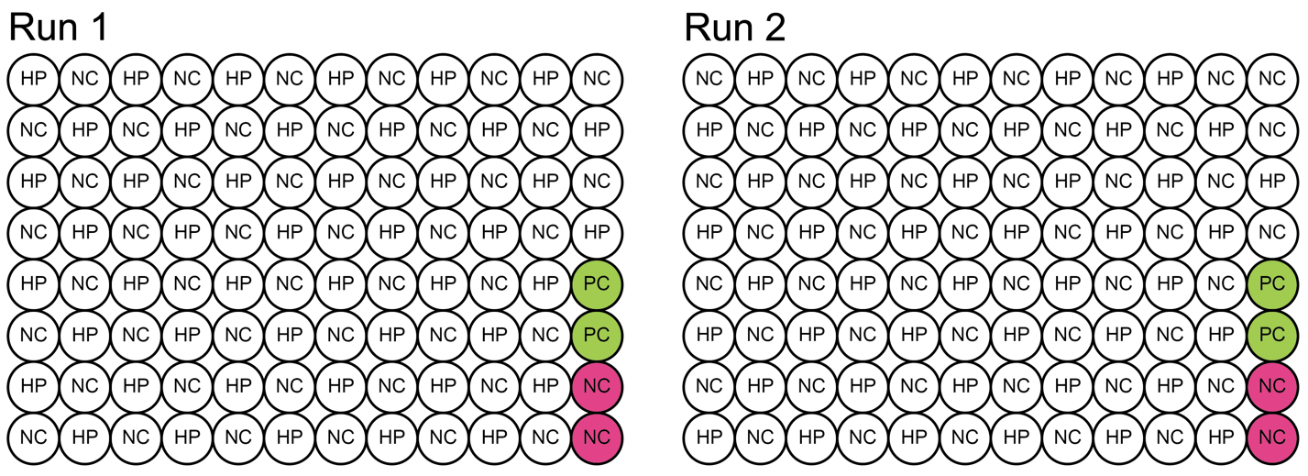


**Supplemental Tables**

**Supplemental Table 1. Reagents used in this study**

| **Reagent** | **Supplier** | **Catalog number** |
| --- | --- | --- |
| LyseNtact buffer (500 mL) | BioEcho | 012-112-500 |
| Warmstart Mastermix | NEB | NEB E1700L |
| Guanidine | Sigma-Aldrich | G4505-500G |
| LAMP Fluorescent dye  HS#38220000 | NEB | NEB B1700S |
| Mineral oil | Merck Life Sciences | M8662-5VL |
| DNase free water | Invitrogen | 10977035 |
| Positive control | Zeptometrix | NATSARS(COV2)-ERC |
| Negative control | Zeptometrix | NATSARS(COV2)-NEG |

**Supplemental Table 2. Equipment used in this study**

| **Equipment** | **Supplier** | **Serial number** |
| --- | --- | --- |
| Biomek FX liquid handler | Beckman Coulter | SN 5012382 |
| Centrifuge Heraeus  Multifuge3LR | Heraerus/Thermo | Model D-37520 sn75004370 |
| LVL decapper | LVL Technologies | SN023083 |
| LVL push cap mat sealer | LVL Technologies | SN210612 |
| LVL tube rack scanner | LVL Technologies | SN M20401 |
| LVL single tube scanner | LVL Technologies | SN D50502 |
| LVL scanner software | LVL Technologies | DataPaq 3.19 (build 3373) |
| Inheco TEC control | Inheco | sn4752 pn8900030 |
| Inheco CPAC unit | Inheco | sn7228 pn7000166-A |
| Inheco TEC control | Inheco | sn1022 pb8900033-A |
| PlateLoc (model 01867-202) | Agilent | SGS11PLC22701 |
| Certus dispenser | Gyger | n/a (multiple dispensers used) |
| Plate centrifuge | Heidolph Titramax 1000 | 100105270 |
| Plate shake Titramax 1000 | BMG | 430-1279/902-0500 |
| Pherastar | BMG | 472-0183/472-0184 |
| Desealer XPeel | Brooks | 10094 |

**Supplemental Table 3. Oligo sequences used for LAMP.**

| **Oligo name** | **Sequence 5’-3’** | **Target** |
| --- | --- | --- |
| Orf1ab_F3^^[[1]](#footnote-1)^^ | CGGTGGACAAATTGTCAC | SARS-CoV-2 Orf1ab |
| Orf1ab_B3 | CTTCTCTGGATTTAACACACTT | SARS-CoV-2 Orf1ab |
| Orf1ab_LF | TTACAAGCTTAAAGAATGTCTGAACACT | SARS-CoV-2 Orf1ab |
| Orf1ab_LB | TTGAATTTAGGTGAAACATTTGTCACG | SARS-CoV-2 Orf1ab |
| Orf1ab_FIP | TCAGCACACAAAGCCAAAAATTTATTTTTCTGTGCAAAGGAAATTAAGGAG | SARS-CoV-2 Orf1ab |
| Orf1ab_BIP | TATTGGTGGAGCTAAACTTAAAGCCTTTTCTGTACAATCCCTTTGAGTG | SARS-CoV-2 Orf1ab |
| RNaseP-POP7-F3 | TTGATGAGCTGGAGCCA | Human RNaseP |
| RNaseP-POP7-B3 | CACCCTCAATGCAGAGTC | Human RNaseP |
| RNaseP-POP7-LF | ATGTGGATGGCTGAGTTGTT | Human RNaseP |
| RNaseP-POP7-LB | CATGCTGAGTACTGGACCTC | Human RNaseP |
| RNaseP-POP7-FIP | GTGTGACCCTGAAGACTCGGTTTTAGCCACTGACTCGGATC | Human RNaseP |
| RNaseP-POP7-BIP | CCTCCGTGATATGGCTCTTCGTTTTTTTCTTACATGGCTCTGGTC | Human RNaseP |

**Supplemental Table 4. Temperature profiles of selected wells in a 384-well flat bottom plate.**

No significant differences were observed between wells A1, L1, A13 and L13 over time. A temperature of 63°C was reached after 6:30 minutes, 64°C after 7:30 minutes and 65°C after 9 minutes respectively.

| **Time in min:sec** | **Well A1** | **Well L1** | **Well A13** | **Well L13** |
| --- | --- | --- | --- | --- |
| 0:00 | 20°C | 20.5°C | 22°C | 22°C |
| 0:30 | 34°C | 35°C | 43°C | 46°C |
| 1:00 | 39°C | 40°C | 47°C | 50°C |
| 1:30 | 43°C | 44°C | 50°C | 53°C |
| 2:00 | 47°C | 47°C | 52°C | 54°C |
| 2:30 | 50°C | 50°C | 54°C | 56°C |
| 3:00 | 53°C | 52°C | 56°C | 57°C |
| 3:30 | 55°C | 55°C | 57°C | 58°C |
| 4:00 | 57°C | 57°C | 59°C | 59°C |
| 4:30 | 59°C | 58°C | 60°C | 60°C |
| 5:00 | 60°C | 60°C | 61°C | 61°C |
| 5:30 | 61°C | 61°C | 62°C | 62°C |
| 6:00 | 62°C | 62°C | 63°C | 63°C |
| 6:30 | 63°C | 63°C | 63°C | 63°C |
| 7:00 | 63°C | 63°C | 64°C | 64°C |
| 7:30 | 64°C | 64°C | 64°C | 64°C |
| 8:00 | 64°C | 64°C | 65°C | 65°C |
| 8:30 | 65°C | 65°C | 65°C | 65°C |
| 9:00 | 65°C | 65°C | 65°C | 65°C |
| 9:30 | 65°C | 65°C | 65°C | 65°C |
| 10:00 | 65°C | 65°C | 65°C | 65°C |
| 10:30 | 65°C | 65°C | 65°C | 65°C |
| 11:00 | 65°C | 65°C | 65°C | 65°C |
| 11:30 | 65°C | 65°C | 65°C | 65°C |
| 12:00 | 65°C | 65°C | 65°C | 65°C |
| 12:30 | 65°C | 65°C | 65°C | 65°C |
| 13:00 | 65°C | 65°C | 65°C | 65°C |
| 13:30 | 65°C | 65°C | 65°C | 65°C |
| 14:00 | 65°C | 65°C | 65°C | 65°C |
| 14:30 | 65°C | 65°C | 65°C | 65°C |
| 15:00 | 65°C | 65°C | 65°C | 65°C |
| 15:30 | 65°C | 65°C | 65°C | 65°C |

**Supplemental table 5. Sensitivity testing of the system for SARS-CoV-2 with External Quality Diagnostics (EQA) panel (QXMD).** Analytical sensitivity was evaluated using Qnostics SARS-CoV-2 Analytical Q Panel (SCV2AQP01-A).

| **Sample code** | **Copies/mL** | **Incubator** | |
| --- | --- | --- | --- |
|  |  | **CPAC** | **CFX** |
| SCV2AQP01-S01 | 1,000,000 | 4/4 | 4/4 |
| SCV2AQP01-S02 | 100,000 | 4/4 | 4/4 |
| SCV2AQP01-S03 | 10,000 | 4/4 | 4/4 |
| SCV2AQP01-S04 | 5,000 | 4/4 | 4/4 |
| SCV2AQP01-S05 | 1,000 | 4/4 | 2/4 |
| SCV2AQP01-S06 | 500 | 2/4 | 3/4 |
| SCV2AQP01-S07 | 100 | 0/4 | 0/4 |
| SCV2AQP01-S08 | 50 | 0/4 | 0/4 |
| SCV2AQP01-S09 | Negative | 0/4 | 0/4 |

1. The *Orf1ab* oligo sequences were adapted from Rabe and Cepko et al.,2018 and the RNase P sequences from Curtis et al., 2018. [↑](#footnote-ref-1)
